# Student Teamwork During COVID-19: Challenges, Changes, and Consequences

**DOI:** 10.1177/1046496420985185

**Published:** 2021-04

**Authors:** Jessica L. Wildman, Daniel M. Nguyen, Ngoc S. Duong, Catherine Warren

**Affiliations:** 1 Institute for Cross Cultural Management, Florida Institute of Technology, Melbourne, USA

**Keywords:** teamwork, COVID-19, pandemic, qualitative, virtual groups, communication, performance

## Abstract

The COVID-19 pandemic has greatly affected all of society, including teams in organizational settings. Collaborative teamwork is particularly susceptible to pandemic disruptions, as coordination across individuals becomes challenging in socially distanced and virtual contexts. Unfortunately, COVID-19 research thus far has primarily studied individual health and performance. Analysis of 90 open-ended survey responses gives voice to students working in project teams during the pandemic and provides future research directions regarding the multilevel impacts of the pandemic on teamwork. Results reflect three themes: (1) challenges experienced; (2) changes to team communication, tasks, and roles; and (3) consequences to team progress and outcomes.

Global crises like the COVID-19 pandemic affect all aspects of society, including collaborative educational and organizational settings. Research suggests that the unexpected but nearly ubiquitous shift online in both employment (Brenan, 2020; Brynjolfsson et al., 2020) and educational settings (Means & Neisler, 2020) has serious impacts on stress, wellness, and satisfaction for individuals attempting to achieve their goals online. However, the focus of most research thus far has been on the individual consequences of the pandemic in terms of work productivity and mental health (e.g., Gao et al., 2020; Luchetti et al., 2020; Rettie & Daniels, 2020; Sun et al., 2020). What has received less research attention is how crisis-induced shifts to virtual work impact the processes of collaboration and teamwork, despite calls for research focused on the pandemic’s impact on the dynamics and performance of what are now almost exclusively virtual teams (e.g., Kniffin et al., 2020).

The lack of research examining the impact of the pandemic on teamwork is problematic because team-based work structures are increasing in prevalence and importance in today’s organizations. Organizational teams are used to address complex problems (Kozlowski & Bell, 2013), and often used in educational settings to improve learning outcomes and ultimately prepare students to engage in effective teamwork in the workforce (Haller et al., 2000; Kalliath & Laiken, 2006). Additionally, teamwork is especially susceptible to the impacts of a pandemic, as teamwork often involves face-to-face interaction and coordination across people, time, and space, making social distancing and virtual work a dramatic shift away from typical teamwork contexts. Accordingly, the current qualitative study aims to give voice to individuals working in teams who experienced the rapid shift to online work triggered by the COVID-19 pandemic, and to use these perspectives to develop directions for future research in this area. To accomplish this objective, we analyze open-ended responses from a sample of undergraduate students working in long-term project teams within upper-level undergraduate courses to answer the following research question:

RQ: What are the perceived impacts of an unexpected pandemic-induced transition to online-only work on team processes and performance in long-term student project teams?

## Sample and Procedure

We solicited responses from student team members working in long-term (i.e., sixteen or more weeks) project teams within upper-level undergraduate courses. Projects included capstone engineering design projects that involved multiple phases such as developing requirements, iterative design reviews, physical fabrication of systems, and testing of systems against requirements, as well as psychological research projects. In response to the COVID-19 pandemic, all in-person classes and interactions were suspended following Spring Break in March of 2020, after which teams worked virtually through the conclusion of the semester in early May of 2020. Embedded within a larger ongoing data collection effort, the following open-ended question was asked both in March and May of 2020: “Given the recent shift of classes from face-to-face to online formats, how have interactions and work processes for your team changed? For example, have you changed how you meet? When you meet? How you communicate? Please describe any changes in as much detail as possible.”

The survey was sent to 259 team members; responses were deleted for any participants that did not respond to the question in either survey, resulting in 95 participants that responded at least once. We then deleted any responses that did not provide substantive information beyond stating modes of communication (e.g., “we use Zoom now”) resulting in a final dataset of 90 responses from 65 participants. Initially, responses were sorted by date in order to explore the extent to which individual’s responses changed between March and May, but there was little discernible change within persons over time, so data were considered holistically. The final sample included 65 participants, with 29 men, 21 women, and 15 participants who did not identify their gender. Participants identified themselves as White (*n* *=* 33), Black (*n* *=* 6), Latinx (*n* *=* 6), Asian (*n* *=* 7), and Native American (*n* *=* 1), while 12 participants did not identify their race. The participants’ average age was 21.88 years old (*SD* *=* 2.83), ranging from 18 to 33 years of age, with roughly a quarter of the participants being international students originally from outside the United States (*n* *=* 15).

## Thematic Analysis

For analysis, we followed Braun and Clarke’s (2006; 2012) thematic analysis steps, including the 15-point checklist of criteria for good thematic analysis. The first author read all comments to get familiar with the data, then went through all comments and made broad initial codes (notes) before iteratively reviewing, grouping, comparing, contrasting, and clarifying the labeling on those initial codes to form larger themes that best represented the responses. Once a set of interpretable and distinguishable themes was identified, each response was revisited to connect them back to the newly formed themes. Also, during this stage, the first author made decisions on what themes to combine, separate, and adjust. For example, the initial codes of *progress slowed* and *progress halted* were combined into the *progress disruptions* theme. In another example, it was determined that all responses could be coded in terms of whether the overall impact on the team was described as negative, neutral, or positive, even though this code was not initially made on all responses. Several initially distinct codes identifying changes in how team members communicated (e.g., less efficient communication, communication perceived as equally effective) were combined into sub-themes within a broader communication change theme. The initial code of *task chang*e was separated into *task chang*e and *role change* to differentiate between changes to the required taskwork of the team versus changes to the roles filled by each of the team members. Motivation loss and morale loss were combined into one category.

## Results

**Figure 1.**
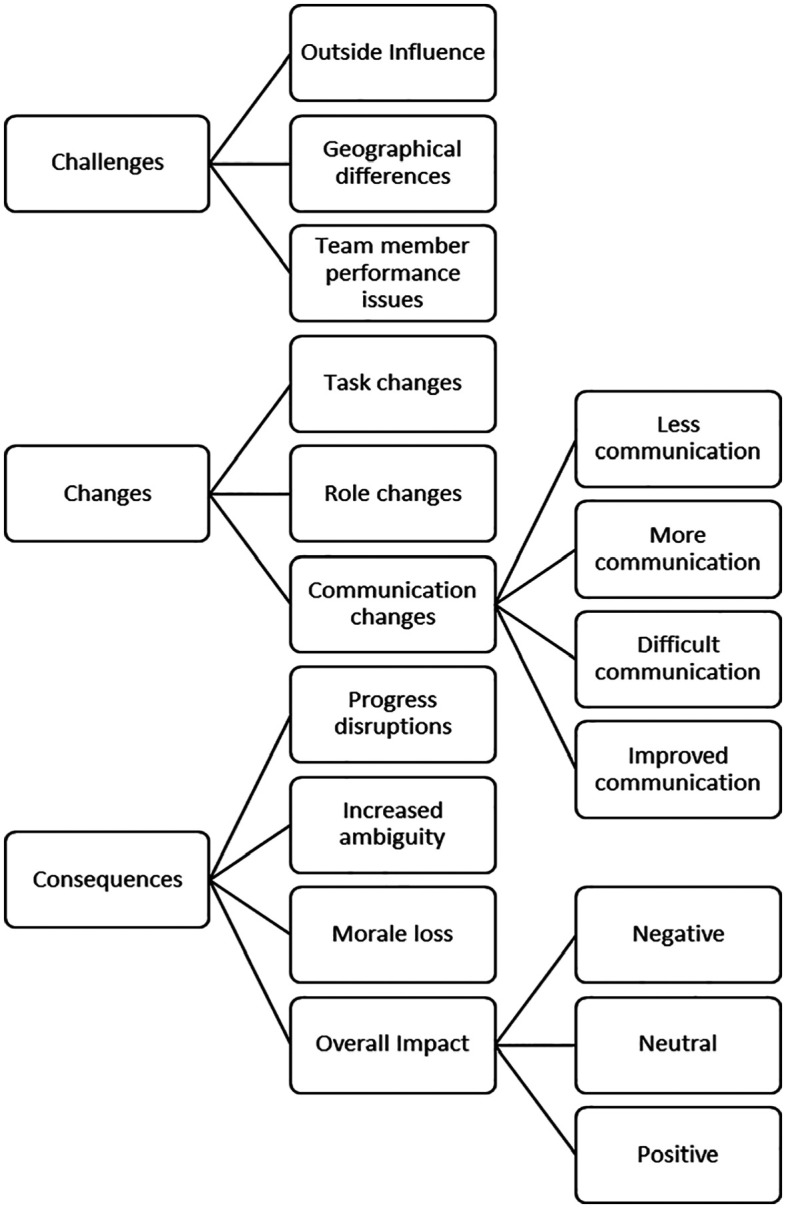
Challenges, changes, and consequences for student teamwork during COVID-19.

The comments analyzed reflect three themes aligned with the initial research question: (1) challenges, or specific obstacles or problems, experienced by teams as a result of the shift to online teamwork during the pandemic, (2) changes, either forced or discretionary, that the team experienced either as a result of the shift online or due to the challenges encountered, and (3) consequences of the shift online to the overall progress and outcomes of the team (Figure 1). Next, we describe the sub-themes within these three categories in more detail and discuss the interrelated implications, limitations, and future research directions drawn from these themes.

### Challenges

Not unexpectedly, many team members reported a variety of challenges that came about during the pandemic that made it difficult to engage in effective teamwork. The first set of teamwork challenges stemmed from the impact of *outside influences*. In other words, these were challenges that impacted the team’s ability to coordinate, communicate, and collectively achieve shared goals, but the primary source of the challenge was external to the team. For example, one participant mentioned increased distractions diverting attention away from the team’s goals caused by working in a home environment:

This is also especially difficult because I am sharing my computer with my mom who is also a teacher and needs to teach her classes, and with my sister who is in high school and needs to meet with her teachers as well. I am extremely worried about my grades now and being able to complete the project in general.

Similarly, another participant mentioned being distracted because of competing demands stemming from other classes (e.g., “but we are also facing plenty of difficulty with other classes which is affecting how much we can put into this”).

Additionally, participants mentioned two other types of challenges. The first is *geographical differences*, which describes some team members’ inability to travel back to campus from various geographical locations due to stay-in-place orders. These changes in the physical context of the team can affect the temporal context (e.g., meeting schedules, project timelines) as well. For example, one participant reported having to change meeting times because team members were now spread across multiple time zones: “We have had to find earlier times to meet which accommodate people being in different time zones.” This is a uniquely team-level challenge in that individuals working independently in different time zones do not need to coordinate as much across time zones. However, because teamwork often requires synchronous meetings and communication, teams are forced to find meeting times that allow all team members to attend, or in some cases, that require some portion of the team to be working during non-business hours. Although this is a challenge that has been well documented in global virtual teams (e.g., Nurmi, 2011), what is notable here is that the pandemic forced a rapid shift from working in collocated face-to-face teams to working in virtual team contexts, which those teams were unlikely to be prepared for.

The second challenge mentioned was *team member performance issues*, which included examples such as perceived increased forgetfulness of team members, increased procrastination within the team, exacerbated issues surrounding social loafing, and an increased need for self-management of the team since there were no longer regularly scheduled class times that forced a minimal level of interaction. For example, one team member described (e.g., “One of our members was non-responsive for 3 weeks so we had to adjust to that.”) In another example, a team members stated, “People seem to forget meeting times more often than when it was face to face.” Performance issues like forgetfulness are not unique to teams, in that research has already highlighted the negative impacts of the pandemic on individual stress and mental health (e.g., Rettie & Daniels, 2020). However, the current research emphasizes that issues caused by suddenly changing to working remotely impact all team members, which will have an impact on the team’s ability to achieve shared goals. Thus, it is important to consider the negative impacts of the pandemic, which can have bottom-up emergent influences not just on the individual directly experiencing the challenge, but on the rest of their team as well.

### Changes

A common change that occurred in response to the rapid shift online, other than utilizing new modes of communication (e.g., Slack, Zoom, WhatsApp), were *communication changes* in terms of both quantity and quality. What is most striking regarding changes to communication is that the changes spanned the continuum: some teams communicated more, some less, and some had no or little change. Some team members mentioned perceiving *little impact* on their team’s communication, especially if they were already using virtual communication modes prior to the pandemic, such as in existing geographically dispersed teams or multidisciplinary teams: “Even when face to face mainly the group communication was through WhatsApp. I am from a different major, so I didn’t see my group if it wasn’t for meetings. Therefore, communication through WhatsApp was crucial.” Another team appeared to not be taking advantage of classroom time for face-to-face interaction even though they could have, and instead had been relying on texting from the start: “However our communication has not changed, we never really talked in class and just texted which is what we still use.” This example is intriguing because it highlights the fact that teams that are technically co-located, if self-managed, can and do opt to interact using virtual means anyways. While it is possible that this choice could have put the team as a disadvantage compared to teams meeting face-to-face at first, it became an advantage in terms of adapting to the pandemic because shifting online was not a disruption to this team’s workflow.

Some team members described having *less communication* during the pandemic. Some teams lost communication with all or part of the team: “The change to online classes has been very rough. Currently there are minimal meetings with team members and communication is low at the moment.” Other teams described having *more communication*, usually because more communication was determined to be necessary to clear up ambiguity: “I think we communicated more because we had to make sure everyone knew what the project was, who did what, and reviewing the weak and strong points of the project without being able to physically meet.” Moving beyond the frequency of communication, many team members describe more *difficult communication*. For some teams, communicating with the entire team became difficult because certain members were hard to get a hold of: “With some group members, it has been easy to communicate, but since we do not see each other in class it is hard to get other group members to respond and contribute to the project.” For other teams, even when communication occurred, clarity was harder to establish: “it is a lot harder to communicate clearly,” or the communication now seemed to be a waste of time: “Communication is not great at this point, meetings seem to be a waste of time in that they all seem to not accomplish anything.”

Finally, some team members experienced *improved communication* as compared to pre-pandemic. These participants perceived that online communication was more efficient or effective than their previous face-to-face communication had been. For example, one participant described the following improvements after shifting online: “Our meetings became more scheduled instead of whenever we were all free which gave more rhythm than before.” Another participant felt the online format was more efficient: “Personally speaking, the online meeting is more efficient than the face to face meeting.” Taken together, the variety of communication changes that occurred in response to the pandemic suggest there may be some unexplored boundary conditions that determine when a team is most likely to respond to external stressors and a sudden shift to online interaction by reducing or increasing communication.

The other team-related changes that occurred in response to the shift online included task changes and roles changes. *Task changes* often occurred because many of these teams were originally engaged in designing physical prototypes, and therefore, the shift online precluded the ability to continue this work. As one team member commented:

As a senior design team, we have completely lost access to the physical portion of our work and will not be able to complete it. We are currently working on rescoping to a project that will still satisfy course requirements without the physical element.

Many teams halted physical work and instead worked on developing the supporting documents for the project: “Now, instead of working hands-on, the team is writing an extra report for the next team that will continue the project explaining what is left to do.” In one case, the project was canceled entirely. This is another uniquely team-level change that was experienced. If an individual was working on a prototype in physical isolation, they likely would have been allowed to continue working on that prototype in both employment or educational settings. However, because these were complex team-based projects, the need to remain socially distanced precluded the teams from continuing their physical work.

Furthermore, *role changes* in terms of the functions fulfilled by team members occurred, sometimes in direct response to the change in taskwork (i.e., the entire project changed, so everyone’s roles changed in response): “Because we no longer meet in person, tasks are assigned to individuals rather than the group as a whole.” In other cases, the team voluntarily chose to make changes to role assignments in response to social loafing or other coordination challenges: “Further we have segmented the team to focus entirely on certain large tasks to split the load and not be held back by interactions required from other people.” In other words, the roles assigned to particular team members often had to change in order to (a) accommodate new taskwork assigned to the team, (b) to accommodate the new virtual working context within which the team was embedded, or (c) in response to team member performance issues. This is another uniquely team-level phenomenon in that shifting roles among team members can only occur within team-based work structures.

### Consequences

Several responses described specific consequences of the shift online during the pandemic for the team’s overall performance. *Progress disruptions* were one of the most immediate consequences for teams such that changes in the project caused forward progress to slow or stop altogether: “The pandemic situation definitely impacted our team negatively. It made us lose the momentum of work.” Some participants also described *increased ambiguity* surrounding the project:

We are kind of lost. We can’t work on the project anymore that we’ve spent the last 2 years of our life on. No closure. The team has no idea what to do next other than busy work and reports etc.

In some cases, this increased ambiguity resulted in changes to communication: “I think we communicated more because we had to make sure everyone knew what the project was, who did what” In other words, for some teams, the abrupt shift online and the changes to the team’s taskwork triggered a need for the team to engage in collective sensemaking in order to reestablish a shared understanding of the project before momentum could be regained. Other participants described *morale loss*, ranging from moderate to very significant, especially in reaction to the loss of the physical design aspect of their projects. For example, one participant described the changes as discouraging: “This is a difficult and discouraging process made worse by lack of in-person contact.” Another described their team as heartbroken:

With our project being ended and competition canceled, the team is pretty heartbroken and the only motivation to finish up our last few papers for the class is the grade. It feels like a lot of hard work for nothing.

A final theme was the *overall impact* of these challenges and changes on the team’s processes and performance. In most cases the impact was *negative*, and in some cases, invoked very strong negative responses: “[Another member] and I have done all of the work since moving online. . .. I absolutely hate being part of this group, it has brought me nothing but stress and added immense amount of work and over-explaining to my course load.” Other participants described changes as quite *neutral*, both in terms of describing a relatively neutral impact and in terms of using relatively neutral language, such as this student in a two-person team:

It has not really changed how I communicate with my team member. We both have a pretty open line of communication and can reach each other pretty easily. The only difference is how we meet. Instead of face-to-face, we now just do zoom meetings in order to talk.

Finally, some impacts were described as *positive* and stated in a positive way: “Our communication is better than it was in person. My group was one of the best I have ever been in. We handled the online switch very well.”

## Discussion

Some of the themes reflected in our data—namely, that the COVID-19 pandemic created a variety of challenges, changes, and consequences for teamwork—are not unexpected. However, these themes do represent one of the first formal research documentation of the phenomenon. Additionally, this research extends previous findings regarding the impacts of the COVID-19 pandemic by finding that performance-impacting issues such as technological limitations, distractions in a home working environment, and an overall increase in stressors, not only impact individuals’ well-being and work performance during the pandemic, but also impact the ability of teams to interact effectively and achieve shared goals.

The implications of the pandemic on performance beyond the individual level are crucial to consider because the interdependent nature of teamwork is likely to exacerbate the impact of any challenges experienced. If the pandemic, or any other jarring global crisis, has a serious impact on an individual working alone, the impact is somewhat limited to that individual’s progress and goal attainment. However, if the pandemic has a serious impact on even one team member within a highly interdependent project team, that impact could trigger a cascade of challenges and changes necessary for the team to recover from the disruption, even if the majority of the team did not directly experience that challenge. The challenges for teamwork only get more complex to manage as more team members are impacted by either the same or different issues (e.g., one has internet connection issues, another has moved to a different time zone, another is suffering from severe anxiety around a family member’s health status) and the team must collectively manage all of these issues at once. In other words, the current research highlights that within teams, challenges are not occurring in a vacuum, and that these challenges could interact with and compound one another. For example, when a team member experiences a challenge while working virtually, it can be difficult for other team members to quickly recognize that assistance or backup behavior is needed, and perceptions of poor performance could trigger tensions and conflict that further negatively impact the team.

The challenges brought about by the COVID-19 pandemic are influencing many different moving pieces at the individual level, and then these individual-level changes are dynamically interacting to impact the team overall. Although the current data shed light on the general concept that micro-level impacts can influence the team’s ability to interact and perform effectively, this research is at the individual level and retrospective in nature, and cannot fully explore the dynamic interactive processes through which higher-level impacts are emerging. Future research should take a microfoundation (Barney & Felin, 2013; Felin et al., 2015) or team microdynamic (Humphrey & Aime, 2014) perspective to explore how the varying impacts of the pandemic on each individual team member, as the diverse constituent elements of the team, interact with one another over time to emerge into a higher-level property of the team that may or may not be functionally equivalent to its lower-level elements (Kozlowski & Klein, 2000; Turner, 1964). Many multilevel emergence-based research questions remain unanswered, such as, but not limited to:

1. Are the impacts of individual team member challenges on the team additive or interactive?
2. Can a positive impact of the pandemic on one team member negate the negative impact of it on another team member?
3. What does the team-level emergent phenomenon look like if each team member is experiencing different challenges versus similar challenges?
4. Is there a critical tipping point at which the team cannot overcome challenges if they are impacting too large a proportion of its team members, or persist for too long?

Another aspect of our research that should be highlighted is that we were specifically studying teams in which the pandemic caused a rapid required shift in work context from face-to-face to virtual, distinguishing it from the ample research on teams that are generally designed from the start to be virtual (e.g., Dulebohn & Hoch, 2017). Therefore, it is important for future research to explore whether this distinction between teams that are designed to be virtual, teams that elect to be virtual, and teams that are abruptly forced to be virtual, has any impact on the interactions and performance of that team. We speculate that the abruptness of this shift online may be one of the boundary conditions that determines when the shift results in process losses versus process gains. Future research should further explore the nature of shifts in virtuality within teams such as the timing of those changes, the level of discretion associated with those changes, and the magnitude or severity of those changes.

In a more hopeful vein, our findings counterintuitively suggested that for some teams, the COVID-19 pandemic had very little or no impact, and in some cases, even a positive impact on teamwork. This is useful to consider in that there is an almost unspoken assumption that the impacts of the pandemic on performance and well-being are overwhelmingly negative. While many negative consequences were reported, there was some evidence for the contrary as well. These themes of neutral and even positive changes and outcomes provide a potential avenue for intervention in teams during crisis-induced shifts to online work. Future research further exploring the reasons why certain teams experienced positive, rather than negative, impacts could help to uncover interventions that could be used to prepare future teams to be more resilient and adaptive in the face of crisis-induced changes. For instance, Stoverink et al. (2020) proposed a model of team resilience based on the conservation of resources theory that may provide additional insights into why certain teams were able to continue interacting effectively despite being exposed to the inherent stressors of the pandemic.

Like any individual study, the current research is not without limitations. First, this research focused on project teams in educational settings, rather than in formal employment settings. However, the project work undertaken in these upper-level undergraduate courses is similar to project work in employment settings: the projects were at least sixteen weeks long and sometimes a year or longer, had clear goals, deadlines, and consequences for success and failure, and the teams were interdependent and multidisciplinary. In other words, there is no reason to expect that these findings would not generalize to other similar types of project teams. However, formal policies and procedures surrounding the COVID-19 pandemic are shifting, and there is ample variance in COVID-19 procedures across educational settings, employment settings, organizations, regions, and nations. Future research should explore the impact of the pandemic on teamwork under varying conditions such as (a) during complete social isolation (i.e., government-mandated stay-at-home orders), (b) during socially distanced and masked in-person interactions, or (c) during unrestricted in-person interactions.

Second, because these data were collected in a rapid timeframe during the COVID-19 crisis, the final sample is relatively small and was subject to extensive nonresponse. Future research should consider the implications of collecting data during global crises, as avoiding nonresponse altogether during such contexts may not be feasible. Regardless, the themes reflected here are most useful for inspiring and informing further future research rather than making direct conclusions. More studies are needed to explore the extent to which these themes are prevalent and generalizable to other samples and organizational contexts. Third, although the focal context of this study was teams, the data are at the individual level and represent individual responses. Therefore, these data cannot speak to whether these perceptions were shared within a team. However, we argue that even if a team member’s perception regarding their team is not shared, it should not be interpreted as not being accurate or meaningful. One team member’s inaccurate or diverging perception can still influence their attitudes and behaviors toward the team and therefore have a bottom-up impact on the team (Ajzen, 1985; Kozlowski & Klein, 2000).

## Conclusion

As society continues to overcome the challenges of the COVID-19 pandemic, the experiences shared by our participants highlight that there are important reasons to research and understand the challenges, changes, and consequences that working teams are facing (see Table 1 for a summary of key insights). Future research should continue to clarify the negative effects of the pandemic on teams, explore how teams can leverage their advantages and experience positive effects, and consider the bottom-up impacts of individual experiences in shaping team-level phenomena.

**Table 1.** Summary of Key Insights.

1. Teams working during COVID-19 suffered from challenges including increased external distractions, forgetfulness, and procrastination.
2. Challenges experienced individually by team members can interact with and compound one another to have an emergent impact on the larger team.
3. Teams working during COVID-19 suffered from challenges unique to teams such as navigating geographical differences between team members and difficulties communicating.
4. COVID-19 changed the communication processes within teams, with some teams communicating less, some communicating more, some having more difficult communication, and some having more efficient communication.
5. Teams engaged in physical work (e.g., prototyping) requiring face-to-face interaction often had to shift to other tasks during COVID-19.
6. Teams often needed to, or chose to, reassign roles among team members in response to the other changes caused by COVID-19.
7. COVID-19 resulted in progress disruptions, increased ambiguity, and loss of morale within teams.
8. Although many of the teamwork impacts of COVID-19 were perceived as negative, some changes (e.g., more efficient meetings) were perceived as positive.
